# Measuring gambling harm in self-reported questionnaires: a scoping review

**DOI:** 10.1186/s12954-025-01352-3

**Published:** 2026-05-24

**Authors:** Cong Mou, Sai Abirami Kailasnathan, Eamonn Ferguson, Richard Tunney, Richard James

**Affiliations:** 1 School of Psychology, University Park, Nottingham, NG7 2RD UK; 2 North London NHS Foundation Trust, 350 Euston Rd., London, NW1 3AX UK; 3 School of Psychology, Aston University, Aston St, Birmingham, B4 7ET UK

**Keywords:** Gambling harm, Measurements, Scoping review, Socioecological framework

## Abstract

**Objectives:**

This review aims to examine how gambling harms are measured by mapping existing self-report measures, analyzing methodologies for scoring gambling harms, and identifying key gaps to inform future research and practice.

**Study design:**

Scoping review.

**Methods:**

The scoping review coded data from 131 studies, analyzing harm measures, corresponding scoring methods, sample-related variables, and associated measures.

**Results:**

(1) This review identified 41 distinct instruments used to measure gambling-related harm over the past two decades, encompassing gambling harm-specific surveys, problem gambling screening tools, custom questionnaires, and questionnaires on harm to affected others. (2) The current landscape reveals that most surveys capture only a subset of harm domains, with dichotomous scoring methods being widely used. (3) Additionally, harm measurement has been largely dominated by a few well-validated instruments, with criterion variables primarily focused on problem gambling severity, gambling engagement, and health-related constructs, and mainly delivered in the global north.

**Conclusions:**

This review highlights discrepancies between conceptual frameworks and real-world practices in the measurement of gambling harm, exposing critical gaps in quantification that have implications for policy decisions, clinical assessments, and public health management. It also questions the generalizability of existing measures to non-WEIRD populations. Future research should prioritize rigorous domain selection, refinement of assessment items, and improvements in quantification methods to better serve the needs of policymakers, researchers, and clinicians. Additionally, establishing cross-cultural validity and utility of gambling harm measures outside of WEIRD contexts is also essential.

**Supplementary Information:**

The online version contains supplementary material available at 10.1186/s12954-025-01352-3.

## Introduction

Gambling-related harm is defined as the negative consequences arising from engagement in gambling activities [1]. This initially focused on adverse impacts experienced as a result of disordered gambling [2]. However, it has gained significant attention in the last decade [3–6], as researchers and policymakers recognized that this overlooked broader harm experienced by lower-risk gambling and affected others. In response, numerous frameworks have been developed to highlight these overlooked perspectives [3, 7–10], emphasizing a socioecological approach integrating individual, interpersonal, and community-level factors. These frameworks underscore the importance of considering these multiple layers—whether for analyzing determinants, designing prevention strategies, shaping policy, or measuring the types of gambling harms.

However, this agreement belies significant disagreement in the practical measurement of gambling harms. One example is the variation in domains emphasized across different surveys. Conceptual frameworks comprehensively categorise these domains, typically including financial harm, work or study-related problems, relationship issues, physical and mental health impacts, cultural or community-level harms, and legal consequences. However, practical surveys may prioritize a subset of these domains. For example, to maximize sensitivity in the general population and to improve data collection efficiency, the Short Gambling Harm Screen (SGHS-10) includes 10 items focused on financial, emotional, and relationship harms, omitting physical health, work-related problems, and broader community impacts [11]. In contrast, tools like the 18-Item Version of the Short Gambling Harm Screen (SGHS-18) and the 7-Item Domain-General Gambling Harm Scale (DGHS-7) attempt to cover as many domains as possible to capture the harm experiences of gamblers with greater severity [12, 13]. The primary aim of this review is to systematically map the harm domains investigated across gambling harm surveys, offering a clearer understanding of the emphasis in current measurement practices and facilitating the identification of critical gaps regarding the assessment of gambling harm domains.

A second inconsistency arises from how responses within the same survey are used, which is closely tied to the conceptualization and quantification of gambling harm. Unlike problem gambling, which is often measured as a categorical construct [14], gambling harm has been conceptualized as a continuous phenomenon that exists along a spectrum of severity [15]. Despite this conceptualization, the field frequently uses pseudo-categorical cutoffs, particularly dichotomous thresholds that indicate the presence or absence of harm [16–20]. Benchmarking studies often justify the continued use of these thresholds, which demonstrate strong associations with decrements in life utility, wellbeing, and heightened mental distress outcomes [6, 19]. This review examines how scoring practices are applied to each measure, and aims to assess the current state of harm quantification in gambling research. We seek to provide insights that can guide the development of future measurement tools better aligned with the needs of stakeholders, including policymakers, researchers, and regulatory bodies.

Measuring gambling harms is a crucial foundation for future prediction and intervention strategies research. As gambling research has shifted from clinical, individual disorder-based models toward public health-oriented frameworks [8, 19, 21], the complex and far-reaching consequences of gambling have become central targets in stakeholder agendas. It is critically important to investigate how gambling measurement approaches reflect these trends and whether current measurement tools adequately support this public health focus. Researchers have continually reflected on its progression and worked to consolidate the knowledge base. Three narrative reviews have been identified, each offering valuable insights. The first review [22] examined early attempts to develop measures targeting gambling harm and more recent approaches focused on low-risk gamblers. It highlighted significant progress in distinguishing gambling harm from problem gambling diagnosis-based measures and achieving greater clarity on the types of harms being measured. The second review [15] evaluated the strengths and limitations of commonly used gambling harm measures, such as the Short Gambling Harm Screen (SGHS-10) and the Domain-General Gambling Harm Scale (DGHS-7). The third review, conducted by Browne and colleagues [23], provided an overview of harm measurement practices over the past 15 years. It categorized measures into four groups: problem-based measures, bespoke instruments, health-economic assessments, and tools specifically designed to assess gambling harm, focusing on SGHS-10. However, we believe that a systematic approach is required to thoroughly investigate how these proposed measures have been applied in practice, beyond the prominent and well-developed scales. Such an analysis is crucial for gaining a comprehensive understanding of measurement trends and identifying gaps. A scoping review is particularly well-suited to this investigation as it allows for a systematic mapping of existing gambling harm measures, coupled with a quantitative synthesis of the data collected from these studies. Given the ongoing debates surrounding the challenges of measuring gambling harms and the continuous efforts to develop new and improved harm measures [22, 23], a scoping review—supported by a comprehensive and systematic search—provides an effective solution to comprehensively examining existing practice that a narrative review cannot do. A scoping review is also preferred instead of a systematic review because there is a need to clarify when a study is using an instrument to measure gambling harm. Previous reviews have noted the use of general scales (e.g., PGSI, DSM) for this purpose [23]. Many studies also make reference to gambling harm despite explicitly measuring gambling disorder or problem gambling. In this review, we aim to investigate how gambling harms are measured in research by synthesizing studies that assess gambling-related harm through data reported by individuals. Specifically, we will examine the harm domains assessed in each measure, the scoring methods applied, and the frequency with which each measure has been used. We will gather psychometric evidence for each measure to better inform future tool selection. Additionally, we conducted exploratory analyses of the sample sizes and the countries from which they were drawn to gain deeper insights into the underlying challenges and to guide future research directions in gambling harm measurement.

## Methods

### Search strategy

For this scoping review, we followed the Preferred Reporting Items for Systematic Reviews and Meta-Analyses extension for Scoping Reviews (PRISMA-ScR) protocol (Supplementary Materials). Studies were identified through searches on Web of Science (WOS), Scopus, APA PsycInfo, and MEDLINE, supplemented by a manual search of references via Google Scholar. The initial search began in September 2023 and was updated on August 8, 2024, using WOS, adding the other three databases on December 09, 2024. A final update was conducted on September 23, 2025, across all databases to capture research published in 2024 and 2025, ensuring the review remained current in this rapidly developing field and relevant upon publication. The search strategy used a combination of abstract terms: (Gambl*) AND (Harm* OR Negative consequence* OR Impact* OR Cost* OR Burden* OR Impairment* OR Damage* OR Adverse effect* OR Negative effect*), with a date range starting from January 1, 2000. Due to the large number of results, an additional filter was applied using”gambling harm” as a keyword for WOS. Non-English papers and certain article types, such as review articles and editorial materials, were excluded.

### Inclusion and exclusion criteria

Only studies that measured gambling-related harms through self-reported questionnaires were included in this review. This criterion also extended to reports and mixed-method qualitative studies, provided they incorporated self-reported harm measures. Studies that assessed harm solely through qualitative methods (e.g., interviews or focus groups) or that discussed gambling harm conceptually without empirical measurement were excluded. Additionally, studies using general outcome measures not directly linked to gambling harm (e.g., wellbeing or quality of life) were omitted. Studies explicitly indicating that they used gambling problem measures (e.g., PGSI [24]) as proxies for gambling harm were included. However, to maintain conceptual clarity, studies that measured only gambling problems without any assessment of harm were excluded.

### Screening

Covidence [25] was used for title and abstract screening, and Zotero [26] was used for organizing the bibliography. The screening process involved two researchers: one conducted a full screening, while the other a quarter of the papers. The agreement percentage for paper inclusion was 94%, and any disagreements were resolved through discussion.

### Data extraction

For each study, we extracted: (1) basic information, including the title, authors, research question, and publication year; (2) sample information, including the study location (country), total sample size, sample characteristics based on gambling frequency and representativeness, gambling activities, sampling methods, gender distribution and the mean and standard deviation of participant ages; and (3) measures, detailing the gambling harm measures used, scoring methods (including cut-off points and their justification, continuous, categorical or binary classification), reliability and validity of the harm measures, and any additional measures used in the study.

### Data processing and analysis

We first identified and sorted the unique measures from the coding results. We then reviewed the questionnaire items to categorize the harm based on the framework proposed by Wardle and colleagues [7]. Ambiguities in domain assignment were resolved through discussion and consensus between two authors.

To enhance the visualization of scoring methods, measures were categorized into four groups based on several issues raised by Browne et al. [23]:

1. The repurposing of problem gambling scales.
2. If the harm was experienced by the gambler or an affected other.
3. The use of a custom or bespoke questionnaire.

The measures were grouped into four categories: “Questionnaires on Harm to Affected Others” (QHAO), “Custom Questionnaires” (CQ), “Problem Gambling Screening Tools” (PGST), and “Gambling Harm-Specific Surveys” (GHSS).

We assessed the geographic distribution of harm measures, identifying the countries where each measure has been applied and the samples involved. Additionally, we combined data across all studies and generated a word cloud to visually represent the most frequently associated measures used alongside gambling harm assessments.

## Results

### Search and selection results

**Fig. 1:**
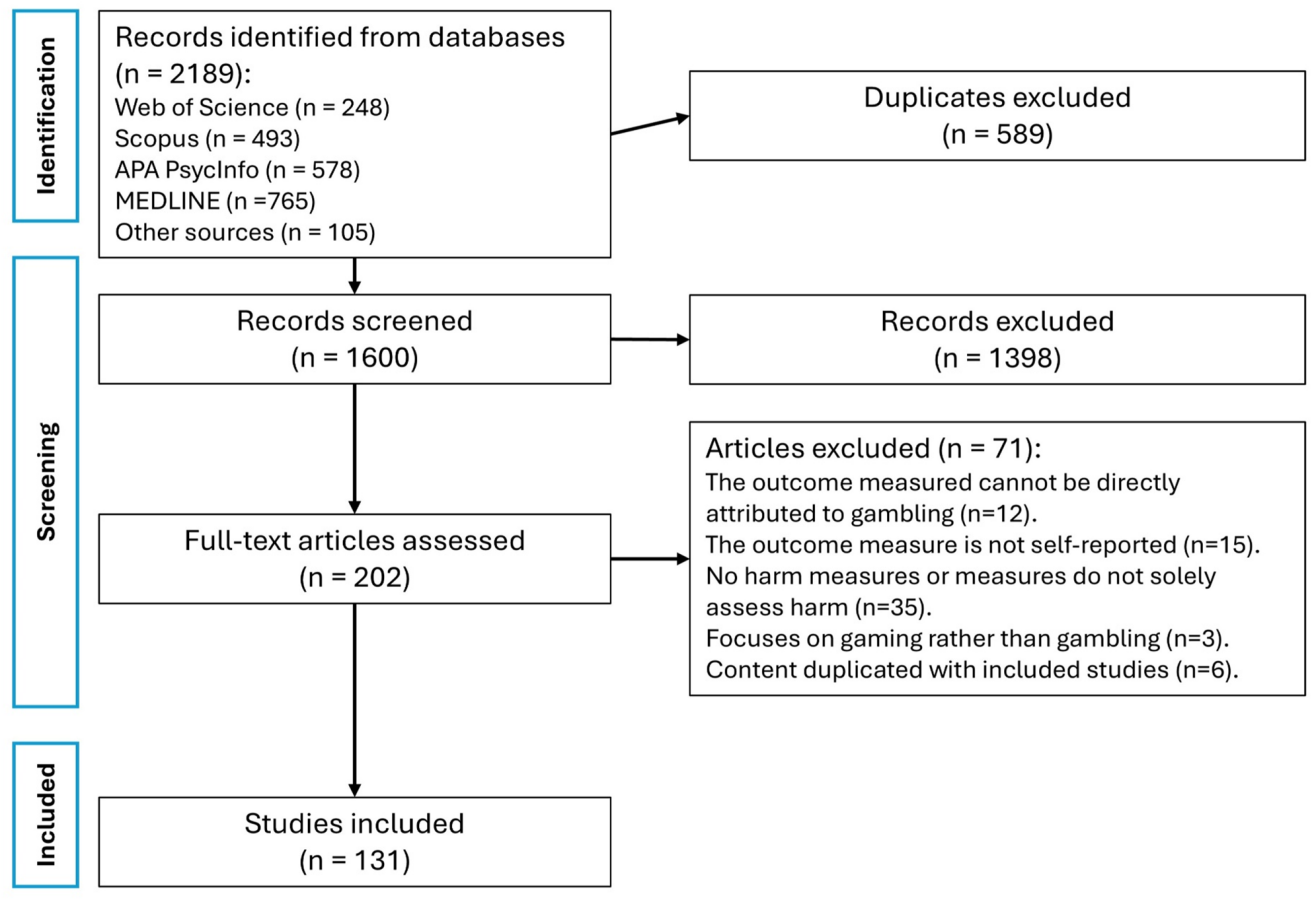
Flow chart of study selection following PRISMA-ScR guidance

The database and manual searches returned 2189 papers for review, with 589 excluded as duplicates. Title and abstract screening led to the exclusion of 1398 papers due to being review articles, commentaries, non-English language publications, or because gambling harm was not a variable of interest. The remaining 202 papers underwent full-text screening, resulting in the exclusion of 71 papers. Finally, 131 studies were included in this review (Fig. 1). The coded data is available in the Supplementary Materials.

### Mapping of gambling harm measures and domains

**Fig. 2:**
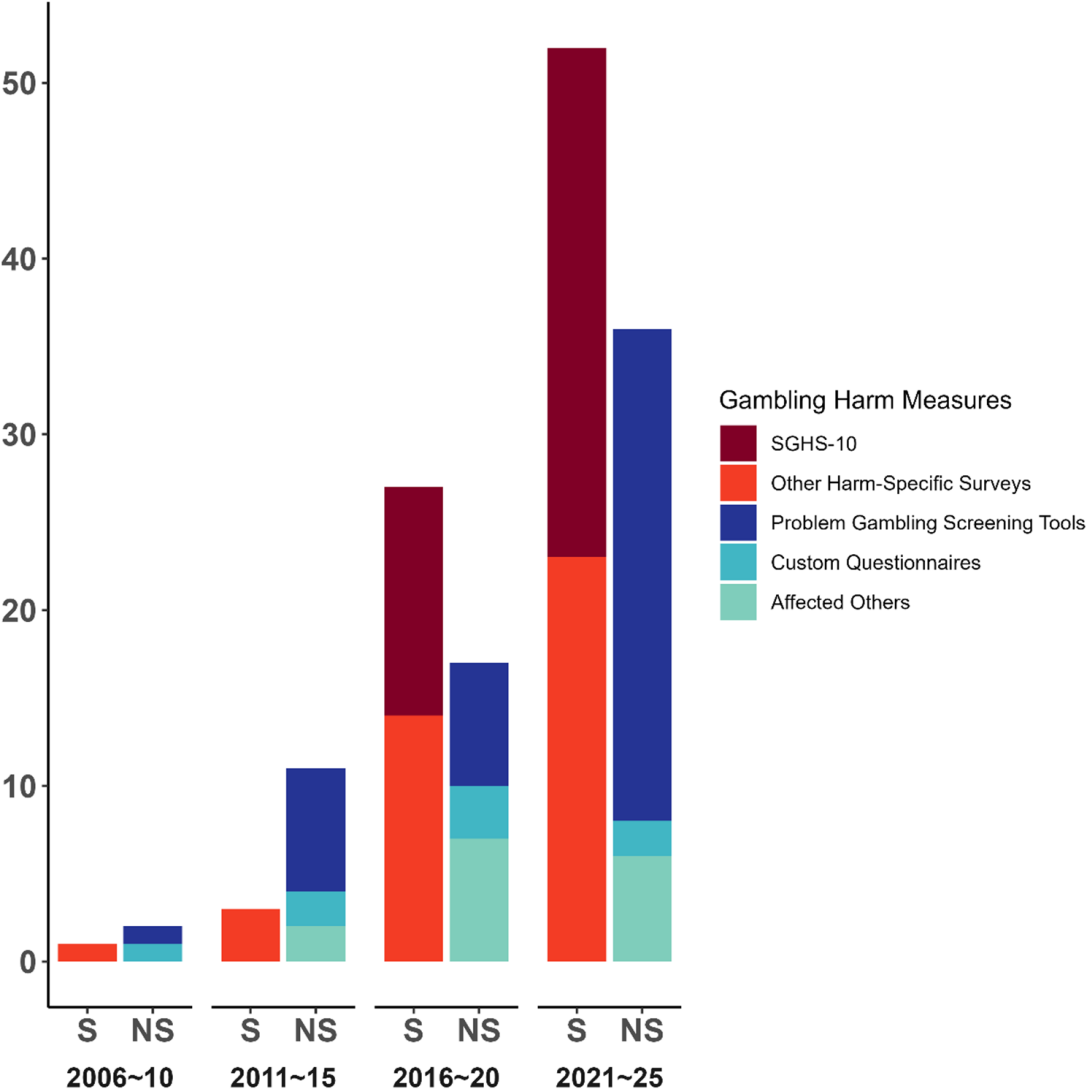
Frequency of measure usage across 5 year intervals. ‘S’ denotes gambling harm-specific measures; ‘NS’ represents other measure groups

Since 2000, 131 studies have used 41 distinct instruments to measure gambling-related harm (Table 1). 16 GHSS, 9 PGST, 8 CQ, and 8 QHAO measures were developed. The frequency of use for each survey is presented in Fig. 2. The results reveal a clear upward trend, reflecting growing interest in gambling harm over time. Notably, the SGHS-10 has seen a sharp rise in popularity since 2015, while the use of problem gambling tools has increased significantly since 2021.

**Table 1:** Summary of gambling-related harms identified in this review and the aspects of harms highlighted according to the items in each measure within the framework proposed by Wardle et al. (2018)

Type	Assessment		Financial			Social		Health	
		n	W	MD	C	PFF	C	PH	MH
Gambling harm specific surveys (GHSS)	Harm questionnaire (HQ)	2	1	1	1	1	1	1	1
	Short gambling harm screen (SGHS-10)	42	0	1	0	1	0	0	1
	Gambling harm measure (GHM)	2	1	1	1	1	1	1	1
	The gambling quality of life scale (GQoLS)	1	0	1	0	1	0	1	1
	18-Item version of the short gambling harm screen (SGHS-18)	3	1	1	0	1	1	1	1
	7-Item domain-general gambling harm scale (DGHS-7)	2	1	1	1	1	1	1	1
	Unimpeachable gambling harms scale (UGHS)	2	1	1	0	1	0	1	1
	Gambling disorder identification test (GDIT)—negative consequences subscale	3	1	1	0	1	0	0	1
	Inventory of consequences scale (ICS) for the gambler (affected others reported)	1	1	1	1	1	0	1	1
	Gamblers inventory of negative consequences (GINC)	1	0	1	1	1	0	0	1
	Victorian framework full harm checklist (GHS)	17	1	1	1	1	1	1	1
	Adolescent harm checklist	3	1	1	1	1	0	1	1
	Consequences due to gambling measure (CDGM)	1	1	1	0	1	0	1	1
	2018 northern territory gambling prevalence and wellbeing surveys harm list	1	1	1	1	1	0	0	1
	Combined gambling harms (CGH)	1	1	1	0	1	0	0	0
	Consequences of gambling problems from gambling impact and behaviour study (GIBS)	1	1	1	1	1	0	1	1
Problem gambling screening tools (PGST)	Canadian problem gambling index (cpgi) harm items (CPGI—harm)^1^	1	0	1	0	1	0	1	1
	South oaks gambling screen (SOGS)	3	1	1	0	1	0	0	1
	NORC DSM screen for gambling problems (NODS)	1	1	1	1	1	0	0	0
	Problem gambling severity index harm items (PGSI—Harm)^2^	11	0	1	0	0	0	1	1
	Problem gambling severity index (PGSI)	22	0	1	0	0	0	1	1
	Victorian gambling screen—harm to self subscale (VGS-HS)	1	0	1	0	1	0	0	1
	GamTest	2	0	1	0	0	0	0	1
	Problem and pathological gambling measure—problems section (PPGM—harm)	1	1	1	1	1	0	1	1
	Diagnostic and statistical manual of mental disorders, 4th edition (DSM-IV) criteria^3^	1	1	1	1	1	0	0	0
Custom questionnaires (CQ)	Harms in jogo do bicho lottery study	1	1	1	1	1	0	1	1
	Financial stress	1	0	1	0	0	0	0	0
	Gambling-related harms in Turkish university students	1	1	1	0	1	0	0	1
	Perceived community impact of problem/pathological gambling	1	1	1	0	1	0	0	0
	Consequences of internet sports betting	1	0	1	0	0	0	0	1
	Negative gambling-related impacts on lifestyle behaviors	1	0	0	0	1	0	1	0
	Gambling harms amongst indigenous australians	1	1	1	1	1	0	0	1
	Impacts of online, mixed, and offline gambling	1	1	1	1	1	0	1	1
Questionnaires on harms to affected others (QHAO)	Gambling harm to children scale	1	0	1	0	1	0	1	1
	Gambling related harms for affected others (GRHs for Aos)	6	0	1	0	1	0	1	1
	20-item gambling harms scale for affected others (GHS-20-AO)	2	1	1	0	1	0	1	1
	Inventory of consequences scale (ICS) for the CSO	1	1	1	0	1	0	1	1
	Family member impact (FMI)	2	0	1	0	1	0	0	1
	Problem gambling significant other impact scale (PG-SOIS)	1	1	1	0	1	0	1	1
	Problem gambling family impact measure (PG-FIM) (gambler reported)	1	0	1	0	1	0	0	1
	Arguments and financial issues in the health and lifestyles survey (HLS)	1	0	1	0	1	0	0	0

n, the number of studies that have used this measure; W, harms affecting work or employment; MD, financial harms related to money or debt; C, crime related harms; PFF, harms affecting partners, family or friends; C, harms to wider community; PH, harms impacting physical health; MH, harms affecting mental health, or inducing psychological distress

1. The CPGI-harm is identical to the PGSI-harm, except there is an additional item about gambling having caused interpersonal problems from the CPGI that is not in the PGSI

2. The PGSI-harm consists of seven out of 9 PGSI items (items related to needing to gamble with larger amounts of money to get the same feeling of excitement, and between-session loss-chasing are removed)

3. The DSM-IV criteria are not a questionnaire in of themselves, but have been converted into an assessment in the study reviewed

The gambling harm domains assessed by each instrument were categorised based on item content (Table 1). While the original framework distinguishes between psychological distress and mental health, we combined these because most instruments did not adequately differentiate between them. Nearly all scales assessed financial harms, with over 80% including items about relationship and mental health impacts. More than half of the tools measured productivity and physical health impairments. Fewer assessments queried harm from crime, and only five instruments assessed community and societal harms, highlighting a significant gap in this area of harm assessment.

### Scoring methods

**Fig. 3:**
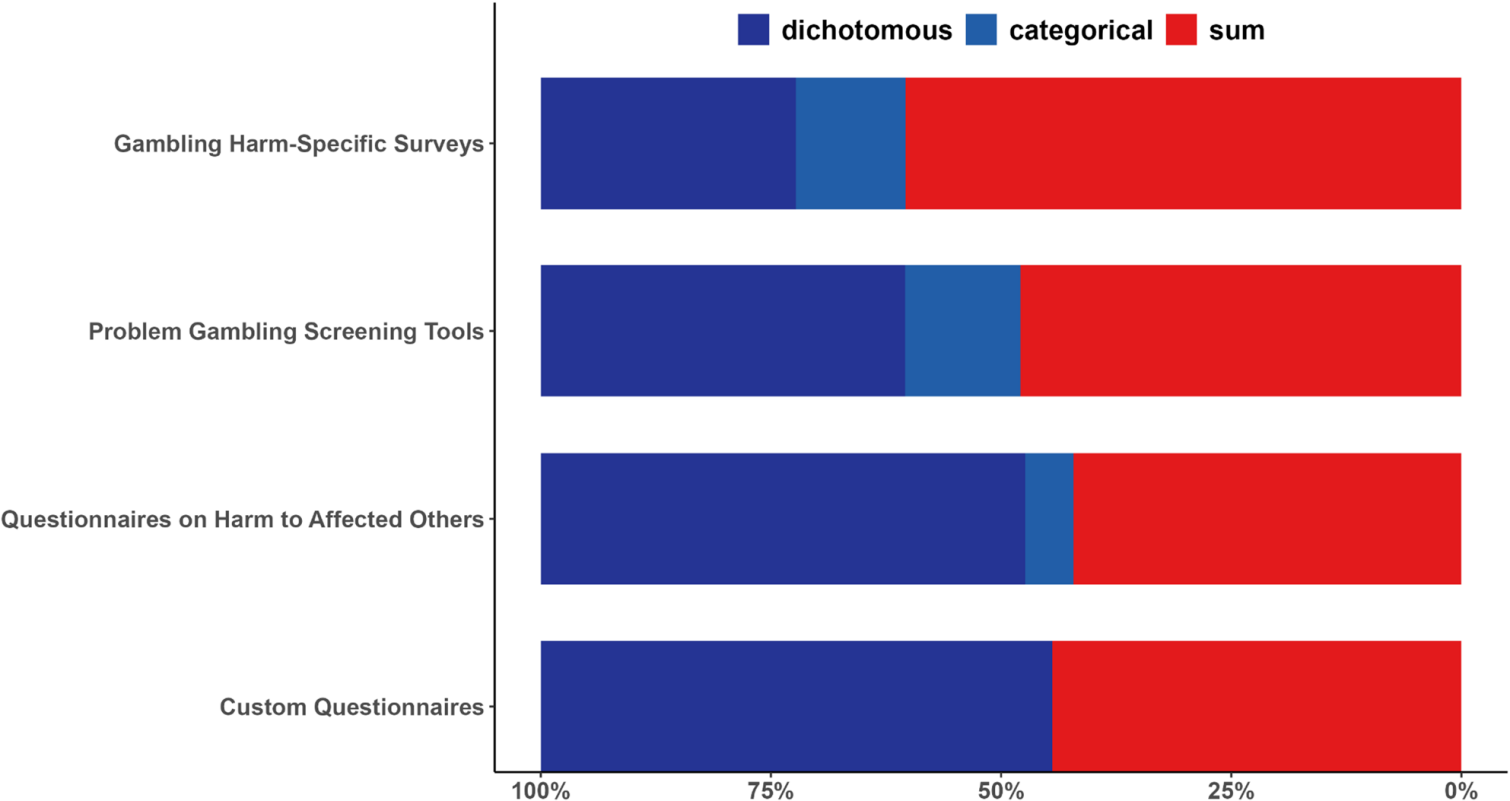
Percentage of scoring methods used in harm measurement groups

Figure 3 shows that GHSS tools predominantly used sum scoring, whereas PGSTs showed a weaker percentage of summing scoring. Notably, across all four categories, dichotomous scoring emerged as a widely used approach, meaning that researchers frequently relied on harm measures to observe the presence or absence of harm—either at the individual item level or across the entire survey. This underscores the prominence of binary classification in gambling harm assessment, showing how harm is identified and interpreted in research.

### Global application, associated constructs, and psychometric validation

**Fig. 4:**
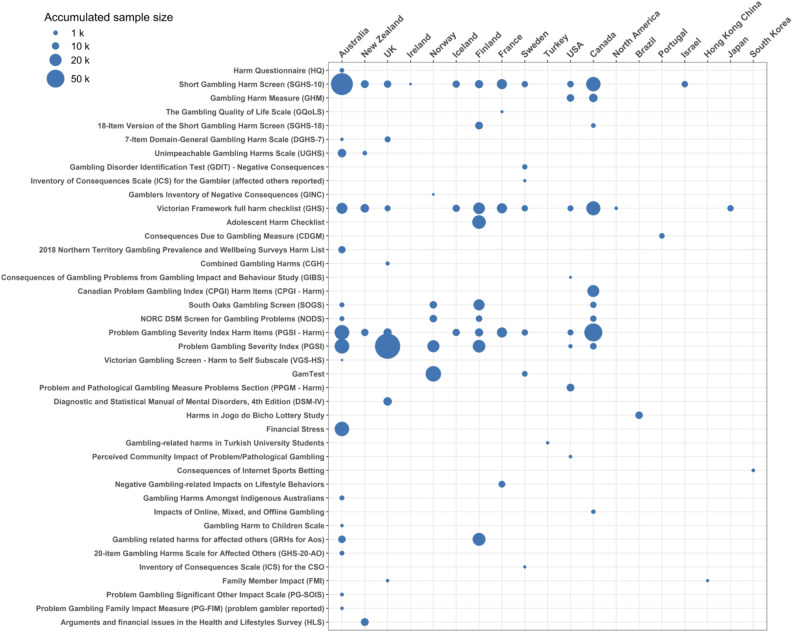
Distribution of countries and sample sizes for each gambling harm measurement instrument

Figure 4 demonstrates that six measures have been applied across at least four countries: the Short Gambling Harm Screen (SGHS-10) [11], the Victorian Framework full harm checklist (GHS) [5], Problem Gambling Severity Index Harm Items (PGSI-Harm) [20], Problem Gambling Severity Index (PGSI) [24], South Oaks Gambling Screen (SOGS) [27], and the NORC DSM Screen for Gambling Problems (NODS) [28]. In contrast, the remaining measures have been applied in only one or two countries, indicating limited cultural reach. Most participant data have been gathered using GHSS and PGST assessments. Few studies have utilized QHAO.

**Fig. 5:**
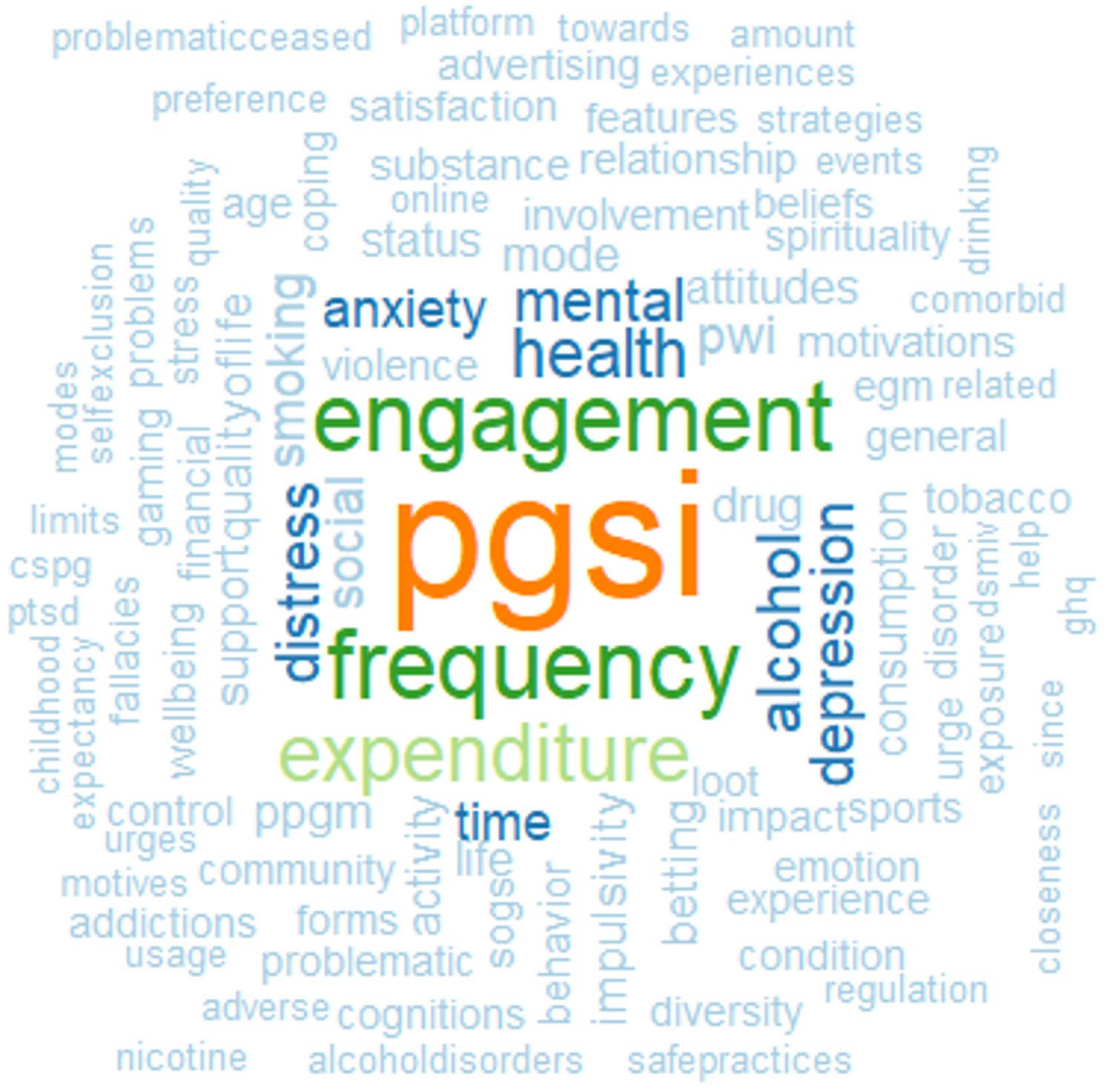
Word cloud depicting gambling harm-related constructs reflected by associated measures

The PGSI was the most frequently used indicator alongside harm measures (Fig. 5). The PGSI frequently served as a validation tool for harm assessment. The second most common were gambling engagement measures such as expenditure and frequency. The third encompassed mental and physical health indicators, including depression, anxiety, disstress, mental health, and comorbid behaviors like alcohol use. These themes collectively illustrate the broader context in which harm measures are applied.

Supplementary Table 1 summarizes the psychometric properties of each harm measure. Many validated harm measures demonstrate strong internal consistency and good convergent validity and are largely characterized by unidimensional factor structures. However, the psychometric validation of QHAO measures is largely unexplored, presenting an area for further research.

## Discussion

The measurement of gambling harm is increasingly important for public health management. This review examined current practices for assessing gambling-related harm. Our scoping review identified 41 distinct measures explicitly claimed to evaluate gambling harm, each with varying target populations, harm domains, and quantification objectives. Two measures, the SGHS-10 and PGSI-Harm, were commonly applied across most countries and the largest sample sizes. This pattern reflects a growing demand for tools that focus specifically on the negative consequences of gambling and highlights the reliance on measures validated in general population samples.

As a formative construct, measuring gambling harm inherently depends on the questions and domains used to capture the intended construct. While there is consensus regarding the core harm domains [3, 7, 8, 15], our findings highlight significant variation in how they are measured and classified. This raises a fundamental question that has been taken for granted in the field for too long—which domains of harm matter most in measurement, and under what conditions? This question has profound policy and research relevance. Without clear measurement priorities, governments cannot adequately assess whether gambling revenues justify their societal costs. Without clarity about the purposes, strengths, and limitations of each measurement tool, researchers risk obtaining suboptimal results and limited insights.

Regarding this question, we propose that no “gold standard” measure will apply to all contexts. Instead, the development and choice of measures must be guided by their intended use, in conjunction with end users such as policymakers, researchers, or public health officials to determine the optimal scale, scope, and focus of harm measurement. For instance, if the primary goal is to monitor the epidemiological prevalence of gambling harm, tools such as the SGHS-10 are well-suited for this purpose as they provide a reliable and sensitive estimate of harm incidence in the general population [5]. Likewise, the PGSI is widely validated and extensively used [19, 28–31], even if the items are intended to be sensitive to categorical thresholds. In this context, comprehensiveness of harm domains is less critical than ensuring that the measure is sensitive and reliable enough to detect harms at lower-risk thresholds [20].

However, if the purpose of harm measurement is to guide resource allocation within public health systems, a comparative approach becomes crucial—one that aligns gambling harm with smoking, alcohol use, and broader mental health disorders. This approach ensures that funding and support structures are equitably distributed. While progress has been made in this direction [32], further research is needed to strengthen the validity and reliability of these cross-sector comparisons [15]. This perspective aligns with the use of macroeconomic analysis for assessing the impact of gambling harms, industry practice, and the potential need for regulation or legislation. Gambling prevalence data, often derived from self-reports of gambling disorder scales, have been used to make assumptions about the breadth and depth of gambling impacts, which are quantified into economic costs. Gambling harm measures have significant potential here, but critical considerations remain. For instance, a measure such as the SGHS-10 will be substantially more informative in sub-threshold cases but may underestimate the degree of harm experienced by the most severe gamblers.

For individual differences research, construct purity and psychometric validation should be prioritized. However, our review reveals striking gaps here—most existing validation studies focus on specific measurement properties. While some research has examined broad temporal trends in gambling harm [33], far less is known about long-term stability, the interplay between different harm domains over time, and how harms propagate across different severity stages. Addressing these gaps will be essential for advancing both theoretical and applied research in gambling harm assessment.

A growing focus on lower-risk gambling and the general population was observed—a defining feature of gambling harm as a public health concern. Unlike diagnostic tools for problem gambling, harm measurement enables a more nuanced understanding of diverse gambling outcomes. It also highlights covert harms such as productivity loss in contrast to more visible consequences like financial difficulties and family conflicts. The next critical step is to refine quantification methods to align with the specific needs of stakeholders. Currently, a significant portion of research validates harm quantification through decrements in life quality or well-being [6, 19]. Although a key strength, this often relies on categorical distinctions (e.g., harm vs. no-harm) when harms are explicitly considered dimensional. The interpretation of some scoring methods remains unclear, particularly regarding what sum scores or harm counts represent and whether counts of severe harms are comparable across individuals. This issue becomes more pressing in clinical contexts where tools like the PGSI or DSM criteria hit ceiling effects. A key advantage of gambling harm measurement is its potential to capture impacts across the full continuum beyond clinical thresholds, combined with quantification methods that account for individual variation in harm severity. This approach could enable more sensitive tools for tracking recovery and informing clinical interventions.

A critical issue of existing gambling harm measures is their limited cross-cultural comparability. Our findings show that most widely used tools were developed and implemented primarily in WEIRD, global north cultures, raising concerns about their relevance in diverse contexts. To promote more accurate and inclusive assessments, future research should prioritize the validation, adaptation, or development of culturally sensitive measures, particularly in regions where gambling is prevalent yet underrepresented [31]. This is especially crucial for capturing culturally specific harms such as stigma or social norm deviations.

This scoping review has several limitations. First, the large number of studies in WOS mentioning gambling harm necessitated using the exact term to refine the search. While this improved specificity, it may inadvertently exclude some studies measuring gambling harm. To mitigate this limitation, we expanded our search across three additional databases without this restriction, conducted manual searches in Google Scholar, and followed citation trails from included studies to identify potentially relevant but omitted research. Second, our review focused on self-reported gambling harm measures. This omits other valuable sources of information about harm, including qualitative reports, administrative data, or behavioural tracking.

Future measurement research should prioritize rigorous justification of domain selection, refinement of assessment items, and enhancement of quantification methods to ensure alignment with the diverse needs of stakeholders. Additionally, greater emphasis is needed on longitudinal studies to advance understanding of the temporal dynamics of gambling harm and its progression over time. Cross-cultural validation in non-WEIRD contexts is equally critical to capture culturally driven variations in harm and determine the need for globally applicable measurement frameworks reflecting diverse social norms and lived experiences.

When employing existing measures, researchers should select harm measures strategically based on the domains and descriptors included, ensuring alignment with the study’s target population—whether general population, lower-risk, or clinical groups. Researchers need to validate harm measures in non-WEIRD contexts before application, particularly for scales including community, religious, or cultural harm items; adapt items as needed to ensure relevance and accuracy.

For public health surveillance, measuring gambling harms over gambling disorder has numerous benefits, including granular evaluation of gambling impacts. One of the contribution of this study is to critically examine existing measures to help researchers and practitioners identify tools most appropriate for their specific public health priorities.

## Supplementary Information

Below is the link to the electronic supplementary material.

Supplementary Material 1.

Supplementary Material 2.

Supplementary Material 3.

## Data Availability

The datasets generated and/or analysed during the current study are available in the OSF repository, https://osf.io/nfmjg/.
